# Real-world outcomes of combined lenvatinib and anti-PD-1 in advanced melanoma: the Lenvamel study, a multicenter retrospective study of the French Group of Skin Cancers (Groupe de Cancérologie Cutanée)

**DOI:** 10.1093/oncolo/oyae145

**Published:** 2024-07-02

**Authors:** Perrine Rousset, Charlée Nardin, Eve Maubec, Valentine Heidelberger, Alexandra Picard, Laura Troin, Emilie Gerard, Nora Kramkimel, Maud Steff-Naud, Gaëlle Quéreux, Caroline Gaudy-Marqueste, Candice Lesage, Claire Mignard, Géraldine Jeudy, Thomas Jouary, Mélanie Saint-Jean, Barouyr Baroudjian, Elodie Archier, Laurent Mortier, Céleste Lebbe, Henri Montaudié

**Affiliations:** Dermatology Department, University Hospital of Nice, Nice, France; Dermatology Department, University Hospital of Besançon, Université de Franche-Comté, Besançon, France; AP-HP, Dermatology Department, Avicenne Hospital, Bobigny, France; AP-HP, Dermatology Department, Avicenne Hospital, Bobigny, France; Dermatology Department, University Hospital of Nice, Nice, France; Dermatology Department, University Hospital of Nice, Nice, France; Dermatology Department, University Hospital of Bordeaux, Bordeaux, France; AP-HP, Dermatology Department, Cochin Hospital, Paris, France; Dermatology Department, CHI Aulnay-Sous-Bois, Aulnay-Sous-Bois, France; Dermatology Department, University Hospital of Nantes, Nantes, France; AP-HM, Dermatology and skin cancer Department, Hôpital Timone, Marseille, France; Dermatology Department, University Hospital of Montpellier, Montpellier, France; Dermatology Department, University Hospital of Rouen, Rouen, France; Dermatology Department, University Hospital of Dijon, Dijon, France; Dermatology Department, University Hospital of Pau, Pau, France; Oncology Department, Institut de Cancérologie de l’Ouest, Saint-Herblain, France; AP-HP, Oncodermatology Department, Saint-Louis Hospital, Université de Paris, Paris, France; AP-HM, Dermatology Department, Hôpital Saint-Joseph, Marseille, France; Dermatology Department, Lille University, Lille, France; AP-HP, Oncodermatology Department, Saint-Louis Hospital, Université de Paris, Paris, France; Dermatology Department, University Hospital of Nice, Nice, France; INSERM U1065, Centre Méditerranéen de Médecine Moléculaire, Université Côte d’Azur, Nice, France

**Keywords:** advanced melanoma, lenvatinib, immunotherapy, anti-PD-1, real-world

## Abstract

**Background:**

Currently, treatment options for patients with advanced melanoma who experience failed immunotherapy or targeted therapy are lacking. Recent studies suggest the antitumor activity of combined pembrolizumab and lenvatinib in patients with advanced melanoma progressing on immunotherapy. Herein, we report the clinical outcomes of combined lenvatinib and a programmed cell death protein-1 inhibitor (PD-1) in this population.

**Materials and Methods:**

This French multicenter real-world study was conducted between September 2020 and July 2023. The primary endpoint was the objective response rate (ORR) according to the Response Evaluation Criteria in Solid Tumours (version 1.1). Secondary variables were treatment-related adverse events (TRAEs), progression-free survival (PFS), overall survival (OS), and duration of response (DOR).

**Results:**

Of the 67 patients included (median age, 69 years; median follow-up, 5.0 months), 85% had stage IV-M1c or M1d disease. The overall ORR was 28.4% (95% CI, 18%-41%), including 3 complete (4.5%) and 16 partial (23.9%) responses. Median DOR was 3.1 (interquartile range, 1.3-4.3) months. Median PFS and OS were 3.1 (95% CI, 2.5-3.7) and 9.8 (95% CI, 5.6-13.9) months, respectively. Grades 3-5 TRAEs occurred in 16 (24%) patients; common TRAEs were fatigue (43.3%), nausea/vomiting (26.8%), diarrhea (20.9%), and hypertension (20.9%). No treatment-related deaths occurred.

**Conclusion:**

Our real-world study demonstrates an interesting response rate and acceptable safety profile in a population with poor prognostic factors. Our data support this treatment option for refractory melanoma, as it is not approved by the Food and Drug Administration or European Medicines Agency, and highlight the need for new strategies.

## Teaser

Treatment options for patients with advanced melanoma who experience failed immunotherapy or targeted therapy are lacking. This article reports the clinical outcomes of combined lenvatinib and a programmed cell death protein-1 inhibitor in this population.

**Implications for Practice**

The Lenvamel study findings consolidate the encouraging results of phase 2 LEAP-004 study by showing an interesting response rate and acceptable safety profile in an immuno-refractory and heavily pretreated population with poor prognostic factors. As it is not approved by the Food and Drug Administration or European Medicines Agency, this treatment may be an option for refractory melanoma, when there is no study available after failure of standard therapies. Patients pretreated with anti-PD-1 plus anti-CTLA-4 and those with *BRAF* wild-type melanoma seemed to benefit more from this strategy. Our study provides promising but limited data on patients with advanced mucosal melanoma.

## Introduction

Over the past decade, the advent of targeted therapy (TT; *BRAF* and *MEK* inhibitors), immune checkpoint inhibitors (ICIs), and inhibitors of programmed cell death protein-1 (PD-1) or its ligand (PD-L1) alone or combined with cytotoxic T-lymphocyte-associated antigen-4 inhibitors (CTLA-4) has considerably improved the survival of patients with unresectable stage III-IV melanoma. Response rates significantly exceed those of chemotherapy, ranging from 40% to 60%–70% in the case of combination therapies, with prolonged responses over time.^1-5^

However, a substantial proportion of patients do not respond to these therapies because of innate or acquired resistance, necessitating additional treatments.^6,7^ Currently, the treatment options for these patients are limited, and new strategies need to be developed.

Some authors have suggested that the multikinase inhibitor, lenvatinib, mainly used in combination with programmed cell death protein (PD)-1 inhibitors, potentiates antitumor activity and overcomes resistance to ICI by inhibiting vascular endothelial growth factor (VEGF) and fibroblast growth factor receptors and shifting the tumor microenvironment to an immune-stimulatory state.^8-13^

Recent studies have suggested antitumor activity of combined pembrolizumab and lenvatinib in advanced melanoma,^14,15^ while lenvatinib monotherapy has demonstrated a poor objective response rate (ORR) of 9%.^16^

In particular, the phase II LEAP-004 study, evaluating combined lenvatinib and pembrolizumab in 103 patients with advanced melanoma progressing on ICI, showed an ORR of 21.4%, reaching up to 33.3% in patients previously treated with anti-PD-1 and anti-CTLA-4 combination therapy. The median duration of response (DOR), progression-free survival (PFS), and overall survival (OS) were 8.3, 4.2, and 14 months, respectively. The safety profile was 46% for grades 3-5 treatment-related adverse events (TRAEs).^14^

The Lenvamel study aimed to investigate the efficacy and safety of lenvatinib combined with anti-PD-1 in a French multicenter real-world patient cohort with advanced melanoma refractory to prior treatment with anti-PD-1 with or without anti-CTLA-4.

## Materials and methods

### Study design and population

This retrospective multicenter study enrolled patients from 16 participating French centers and was conducted between September 2020 and July 2023.

Inclusion criteria were: age > 18 years, diagnosis of unresectable stage III or IV melanoma, treatment with combined lenvatinib and anti-PD-1, and resistance to prior anti-PD-1 therapy (monotherapy or in combination). Patients previously treated with adjuvant immunotherapy were included. Those with primary uveal melanoma were excluded.

We collected the following patient data: age, sex (male or female), disease stage (III or IV according to the American Joint Committee on Cancer [AJCC], 8th edition), melanoma type (cutaneous, mucosal, and unknown), Eastern Cooperative Oncology Group (ECOG) performance status (0, 1, or ≥2), serum lactate dehydrogenase (LDH) level (≤upper limit of normal [ULN], >ULN, <2ULN, or ≥2ULN), *BRAF V600* status (wild type or mutated), *NRAS* and *CKIT* status (wild type or mutated), number of metastatic sites (≤3 or >3), stereotactic radiosurgery (SRS) for active brain metastasis (progressive and/or symptomatic brain metastasis at the initiation of anti-PD-1 plus lenvatinib), number of previous treatments (including ICI, TT, chemotherapy, or others, in adjuvant and/or metastatic setting; 1, 2, or ≥3), and resistance to anti-PD-1 therapy. Primary resistance in the adjuvant setting was defined as follows: (1) progressive disease (PD) within 12 weeks after the last anti-PD-1 therapy dose used in the adjuvant setting, versus primary resistance in the metastatic setting; (2) best stable disease (SD) or PD response to anti-PD-1 (metastatic setting) versus secondary resistance in the metastatic setting; and (3) PD after a previous best complete response (CR) or partial response (PR) to anti-PD-1.

We also collected data on combined treatment with lenvatinib and anti-PD-1, including treatment duration, regimen dose, dose reduction and/or interruption, and toxicity.

In order to ensure replicability, data were collected using a uniform and standardized case report form (CRF). Investigators at each site, reviewed, completed, and validated relevant anonymized patient data in the CRF.

### Treatment regimen

All patients received lenvatinib orally at doses of 20, 14, 10, or 8 mg, once daily. The choice of initial dose and ICI regimen was at the discretion of the physicians at each participating center. Lenvatinib treatment could be interrupted or the dose reduced to manage toxicity.

The concomitant SRS usage data were collected for patients with brain metastasis.

### Assessments

All patients at baseline or just before initiating anti-PD-1 plus lenvatinib were subjected to staging with a thoraco-abdominopelvic computed tomography (CT) or positron emission tomography-CT. Brain imaging and other imaging modalities, such as magnetic resonance imaging, were performed based on clinical indication at the discretion of the treating physician. Then, radiographic assessment was performed every 12 weeks. The response was assessed per Response Evaluation Criteria in Solid Tumours (RECIST) version 1.1, by investigator assessment.^17^ Adverse events (AEs) and laboratory abnormalities were collected regularly during study treatment and graded using the National Cancer Institute Common Terminology Criteria for Adverse Events version 5.0. Adverse events and immune-related adverse advents (irAE) of clinical significance were defined as any immune-breakthrough adverse event that was grade 3 or higher or any event that caused discontinuation or resulted in permanent organ dysfunction. TRAEs and irAE were categorized by symptoms into cutaneous, cardiovascular, endocrinologic, rheumatologic, gastrointestinal, hepatic, pulmonary, hematological, ophthalmological, renal, general, and musculoskeletal.

### Outcomes

The primary endpoint was ORR, defined by radiologists from each participating institution as the proportion of patients with CR or PR according to RECIST v1.1.^17^

Secondary endpoints were PFS, OS, DOR, and safety. PFS was defined as the time from the start of treatment to the date of PD or death, whichever occurred first. Patients who remained alive without PD were censored on the date of their last assessment. OS was defined as the time from the start of treatment to death. Patients who remained alive were censored on the date of last contact. DOR was defined as the time from the first evidence of CR or PR to PD or death in responsive patients. Patients who maintained CR or PR were censored on the date of their last visit. For PD, we collected data regarding the next therapeutic line (continuation of lenvatinib plus anti-PD-1, ICI, TT, CT, palliative care, or others).

### Statistical analysis

Baseline patient characteristics are presented as numbers and percentages for categorical variables, and as mean (standard deviation), median (interquartile range, [IQR]), and range for continuous variables, as appropriate. The Clopper–Pearson method was used to calculate 95% exact binomial CIs for ORR. Patients without response assessments were considered nonresponders. DOR, duration of SD, PFS, and OS were estimated using the Kaplan-Meier method. Patients lost to follow-up were censored at their last visit. Missing data were not replaced.

## Results

### Population characteristics

The 67 included patients had a median age of 69 (IQR, 27-88) years and follow-up duration of 5.0 (IQR, 3.3-9.8) months.

Fifty-seven (85%), 42 (63%), 26 (39%), and 58 (86%) patients had stage IV M1c or M1d disease, ≥3 metastatic sites, elevated LDH levels, and ECOG <2, respectively. Twenty (30%) patients had active brain metastasis. Thirteen (19%) and 20 (30%) patients had *BRAF* V600 mutations and *NRAS*-mutant melanoma, respectively.

Most patients had received ≥3 prior lines of therapy (45%), whereas 58 (87%) had experienced progression on prior anti-PD-1 and anti-CTLA-4 therapy. All patients with *BRAF* V600 mutations were pretreated with targeted therapy.

The anti-PD-1 resistance phenotype showed primary resistance in the adjuvant setting in 8 (12%) patients, and primary and secondary resistance in a metastatic setting in 29 (43%) and 30 (45%) patients, respectively.

All patients received >1 dose of lenvatinib combined with anti-PD-1 (pembrolizumab (200 mg every 3 weeks) for 55 (82%) or nivolumab (480 mg every 4 weeks) for 12 (18%) patients). One patient received concurrent intrathecal nivolumab therapy. Fourteen of the 20 patients (70%) with active brain metastasis received concomitant brain SRS. At data cutoff, 53 (79%) patients had discontinued all treatments, most commonly for radiographic PD in 32 (60%) patients, followed by toxicity in 14 (21%) patients. All demographic and disease characteristics are presented in Table 1.

**Table 1.** Baseline demographic and disease characteristics.

Baseline characteristics	All patients (*N* = 67)
Age (years)	
Mean	64
Median (range)	69 (27-88)
≥65, *N* (%)	38 (57)
Sex, *N* (%)	
Men	33 (49)
Women	34 (51)
Primary melanoma, *N* (%)	
Cutaneous	54 (81)
Mucosal	7 (10)
Unknown	6 (9)
AJCC 8th edition, *N* (%)	
III	2 (3)
IV	65 (97)
M1a	1 (2)
M1b	7 (10)
M1c	28 (42)
M1d	29 (43)
Active brain metastasis, *N* (%)	20 (30)
With concomitant brain stereotactic radiosurgery	14 (70)
Without concomitant brain stereotactic radiosurgery	6 (30)
Number of disease sites, *N* (%)	
≤3	25 (37)
>3	42 (63)
ECOG PS, *N* (%)	
0	23 (34)
1	35 (52)
≥2	9 (14)
Mutations status, *N* (%)	
*BRAF V600*	13 (19)
*NRAS*	20 (30)
*CKIT*	4 (6)
LDH, *N* (%) > ULN; IC (95%)	
Normal	33 (49)
N > ULN and <2ULN	20 (30)
N ≥ 2ULN	6 (9)
Unknown	8 (12)
Number of prior lines of therapy, *N* (%)	
1	19 (28)
2	18 (27)
≥3	30 (45)
Prior line with Ipilimumab + Nivolumab, *N* (%)	58 (87)
First-line treatment, *N* (%)	
Immune checkpoint inhibitors	63 (94)
Anti-PD-1 in adjuvant setting	13 (19)
Anti-PD-1 in metastatic setting	24 (36)
Ipilimumab + Nivolumab	26 (39)
Targeted therapy	3 (4)
BRAFi + MEKi in adjuvant setting	1 (1)
BRAFi + MEKi in metastatic setting	2 (3)
Triplet therapy (Anti-PD-1 + BRAFi + MEKi)^a^	1 (1)
Chemotherapy	0 (0)
Previous line before starting anti-PD-1 and lenvatinib, *N* (%)	
Immune checkpoint inhibitors	50 (75)
Anti-PD-1 in adjuvant setting	2 (3)
Anti-PD-1 in metastatic setting	18 (27)
Ipilimumab + Nivolumab	30 (45)
Targeted therapy	6 (9)
BRAFi + MEKi in adjuvant setting	0 (0)
BRAFi + MEKi in metastatic setting	5 (7)
MEKi alone	1 (1)
Triplet therapy (Anti-PD-1 + BRAFi + MEKi)	1 (1)
Chemotherapy	6 (9)
Others^b^	4 (6)
Resistance to anti-PD-1, *N* (%)	
Primary resistance in the adjuvant setting	8 (12)
Primary resistance in the metastatic setting	29 (43)
Secondary resistance in the metastatic setting	30 (45)
Follow-up (months)	
Mean	7.5
Median [IQR]	5.0 [3.3-9.8]

^a^Clinical trial Combi-I: NCT02967692.

^b^Other therapies were pembrolizumab plus trametinib (*N* = 2), nivolumab plus relatlimab (*N* = 1), sorafenib (*N* = 1).

Abbreviations: AJCC, American Joint Committee on Cancer; IQR, interquartile range; ECOG, Eastern Cooperative Oncology Group; LDH, lactate dehydrogenase.

### Efficacy

Table 2 shows the response rates. The best ORR was 28.4% (95% CI, 18%-41%), with 3 (4.5%) and 16 (23.9%) patients achieving CR and PR, respectively. The median DOR and SD duration were 3.1 (IQR, 1.3-4.3) and 2.3 months, respectively. Estimated SD duration of ≥6 months was seen in 12.5% of participants.

**Table 2.** Response rates.

Total population (*N* = 67)	
Best overall response, no. (%)	
Complete response	3 (4.5)
Partial response	16 (23.9)
Stable disease	8 (11.9)
Progressive disease	32 (47.8)
Not evaluated	8 (11.9)
Objective response, no. (%) [95% CI]	
No. of patients	19 (28.4) [18-41]
Disease control	
No. of patients	27 (40.3)
Duration of response	
No. of patients	19
Median (months)	3.1
Range (months)	0.6-20.8

Among the 19 patients who achieved initial PR or CR, 11 (58%) experienced PD. At data cutoff, 13 (19%) patients remained on treatment, and 1 patient had a maximum follow-up of 18.8 months.

Among the remaining 51 patients who discontinued the treatment for toxicity or PD, 23 (45%) received exclusive palliative care, while 28 (55%) received a new antitumoral therapy: 16 (31%) received CT; 5 (10%), TT; 5 (10%), ICI; and 2 (4%), combined anti-PD-1 and TT.

Regarding prior therapy, ORR was 32.8% (95% CI, 22%-46%) in patients progressing on prior anti-PD-1 and anti-CTLA-4 combination therapy, regardless of the line, and 38.5% (95% CI, 22-57) in those treated with first-line anti-PD-1 and anti-CTLA-4 therapy. ORR was 33.3% (95% CI, 13-59) for patients who received 2 prior lines of therapy and 20% (95% CI, 8-39) for patients who received 3 and more prior lines of therapy. None of the 5 patients who received targeted therapy, nor any of the 2 patients who received adjuvant anti-PD-1 immediately prior to pembrolizumab plus lenvatinib experienced objective response. By resistance phenotype, ORR was 12.5% (95% CI, 2%-47%) for primary resistance in the adjuvant setting, 34.5% (95% CI, 20%-53%) for primary resistance in the metastatic setting, and 26.7% (95% CI, 14%-44%) for secondary resistance in the metastatic setting. By tumor characteristics, ORR was 71.4% (95% CI, 29%-96%) for primary mucosal melanoma versus 24.1% (95% CI, 14%-38%) for primary cutaneous melanoma, and ORR was 31.5% (95% CI, 20%-46%) for *BRAF* wild-type melanoma versus 15.4% (95% CI, 2%-45%) for *BRAF*-mutant melanoma.

By prognostic factors, ORR was 45.0% (95% CI, 23%-68%) for patients with active brain metastasis, 19.0% (95% CI, 10%-33%) for >3 metastatic sites versus 44% (95% CI, 24-65) for <3 metastatic sites, and 30.8% (95% CI, 17%-50%) for LDH levels >ULN.

Supplementary Table S1 shows the response patterns by subgroups.

**Figure 1.**
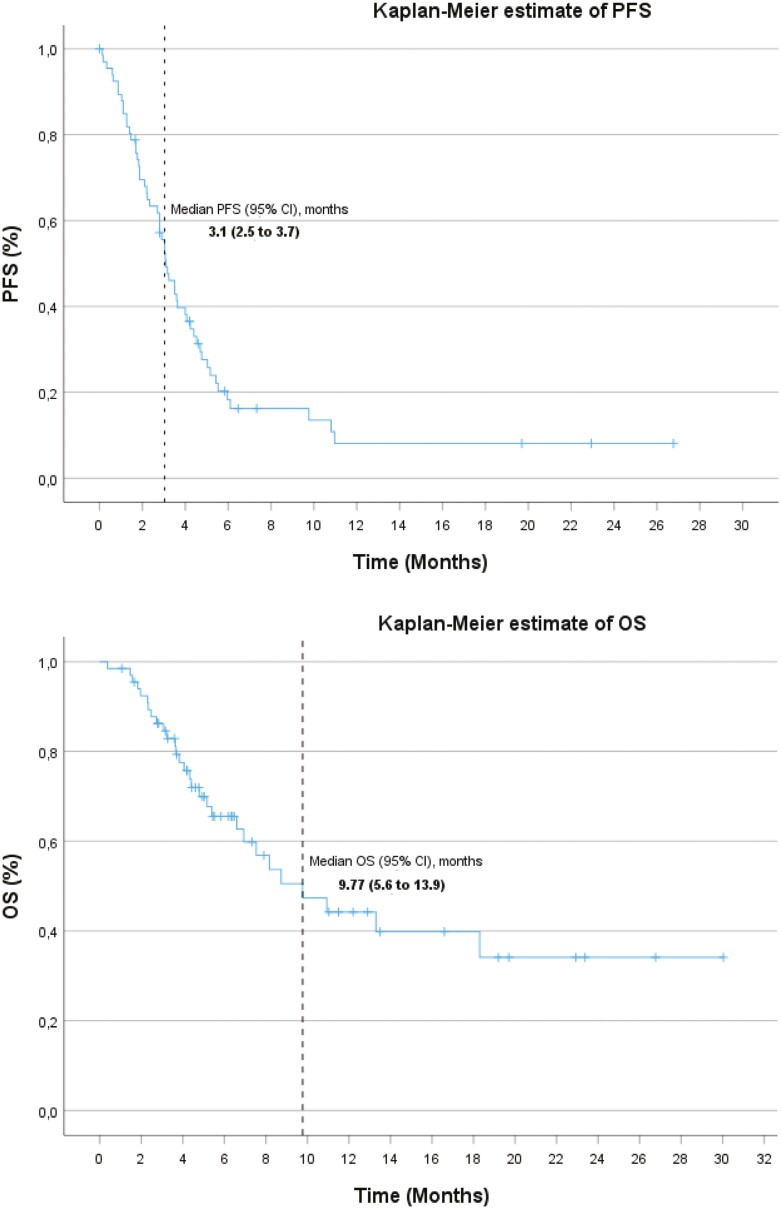
Survival endpoints. Kaplan-Meier estimate of PFS (A) and OS (B). Abbreviations: OS, overall survival; PFS, progression-free survival.

With 55 (82.1%) PD or death events in the total population, the median PFS was 3.1 (95% CI, 2.5-3.7) months, with estimated 6- and 12-month PFS rates of 14.9% and 4.5%, respectively (Figure 1A). With 29 (43.4%) deaths, the median OS was 9.77 (95% CI, 5.6-13.9) months, with estimated 6- and 12-month OS rates of 40.3% and 17.9%, respectively (Figure 1B).

### Tolerance data

The median lenvatinib treatment duration was 2.9 months (range 4 days–22.9 months), with a mean dose intensity of 15.8 mg daily. Eighteen (26.9%) patients underwent at least 1 lenvatinib dose reduction. The median duration of pembrolizumab treatment duration was 3.3 months (range 4 days-22.9 months; Supplementary Table S2).

Fifty-four (81%) patients experienced a total of 183 TRAEs of any grade, and 16 (24%) experienced 25 grade 3-4 AEs (Table 3). At data cutoff, 14 (21%) patients had discontinued all treatments for toxicity.

**Table 3.** Rate and severity of AEs.

Population	Patient No.	Adverse events		
		All grades, No. patients (%)	Grade I-II (%)	Grade III-IV (%)
Total	67	54 (81%)	38 (57%)	16 (24%)
Total TRAEs leading to interruption of lenvatinib, anti-PD-1, or both				
	67	23 (34%)	10 (15%)	13 (19%)
Total TRAEs leading to discontinuation of lenvatinib, anti-PD-1, or both				
	67	14 (21%)	8 (12%)	6 (9%)
Most frequent TRAEs (all cohort *N* = 67 patients)				
AEs	Any grade, No.(%)		Grade III-IV, No.(%)	
Fatigue	29 (43.3%)		5 (7.5%)	
Nausea and vomiting	18 (26.8%)		2 (3.0%)	
Hypertension	14 (20.9%)		4 (6.0%)	
Diarrhea	14 (20.9%)		1 (1.5%)	
Hypothyroidism	9 (13.4%)		0 (0%)	
Anorexia	8 (11.9%)		0 (0%)	
Proteinuria	8 (11.9%)		4 (6.0%)	
Weight loss	7 (10.4%)		1 (1.5%)	
Abdominal pain	7 (10.4%)		1 (1.5%)	
Arthralgia	6 (9.0%)		2 (3.0%)	
Liver test abnormalities	6 (9.0%)		1 (1.5%)	
Stomatitis	6 (9.0%)		0 (0%)	
Dysphonia	5 (7.5%)		0 (0%)	
Thrombocytopenia	5 (7.5%)		1 (1.5%)	
Hemorrhage	2 (3.0%)		0 (0%)	

Most common TRAEs of any grade were fatigue (43.3%), nausea and vomiting (26.8%), hypertension (20.9%), diarrhea (20.9%), and hypothyroidism (13.4%). Other TRAEs of any grade that occurred in ≥5 patients were anorexia (11.9%), weight loss (11.9%), proteinuria (11.9%), abdominal pain (10.4%), stomatitis (9.0%), liver test abnormalities (9.0%), arthralgia (9.0%), dysphonia (7.5%), and thrombocytopenia (7.5%). No treatment-related deaths occurred during the follow-up. The occurrence of AEs of any grade, by the different organs/systems involved, is presented in Supplementary Table S3.

## Discussion

Despite therapeutic advances in the management of advanced melanoma owing to immunotherapy and TT, a substantial proportion of patients experience innate or acquired resistance. Currently, the treatment options for these patients are limited, and new strategies need to be developed. In a phase Ib/II study cohort of 21 patients with metastatic melanoma who received ≤2 previous systemic therapies (62% were treatment-naïve), combined lenvatinib and pembrolizumab showed an ORR of 48%, 12.5-month median DOR, and 5.5-month median PFS.^15^ The phase II LEAP-004 study showed, in a cohort of 103 patients previously treated with anti-PD-1 with or without anti-CTLA-4, that the ORR was 21%, with a median DOR of 8.3 months.

The outcomes in our real-world cohort are similar. Our population showed an ORR of 28.4%. This is a clinically significant rate considering the substantial proportion of patients displaying poor prognostic factors (eg, 43%, 63%, and 39% had stage M1d disease, >3 metastatic sites, and elevated LDH levels, respectively) and heavily pretreated melanoma (including 45% and 87% patients with ≥3 prior therapy lines and anti-CTLA-4 and anti-PD-1 combination pretreatment, respectively). Responses were observed in most subgroups except in patients with LDH levels >2ULN. In patients with active brain metastases, ORR was 45%. This impressive result could be explained in part by the finding of Tran et al, who suggested that lenvatinib may modify brain endothelial permeability and promote trans-endothelial migration of immune cells.^13^ It is noteworthy that 14 (70%) patients underwent concomitant brain SRS. In a recently published smaller cohort, overall ORR was 28% and 31% for patients with brain metastasis. In this study, all the patients with brain metastasis received SRS.^18^

Unexpectedly, no response was observed for patients with stages III and IV-M1a melanoma; however, the sample size was small, with 2 and 1 patient, respectively. In contrast to the LEAP-004 study which suggested a similar likelihood of response across clinical resistance phenotypes, patients with primary anti-PD-1 resistance in the adjuvant setting in our cohort seemed to benefit less from this strategy. However, our results must be interpreted with caution given the small sample size. A deeper understanding of mechanisms underlying resistance phenotypes would allow us to adjust strategies and develop more effective treatments.

Interestingly, ORR was higher in patients who previously received first-line combined anti-CTLA-4 and anti-PD-1 therapy (38.5%), compared to those treated with anti-PD-1 alone (8.3%), similar to the LEAP study results. Response rates were lower in patients with *BRAF*-mutant tumors (15.4%) compared to those with *BRAF* wild-type tumors (31.5%). This lower benefit in patients with *BRAF* mutations has already been reported^14,18^ without specifying whether a link occurred by the cross-pathway resistance mechanisms.^19^

An impressive response was also observed for mucosal melanoma with an ORR of 71.4%. Literature on this subject is limited, but recent studies have highlighted the promising efficacy of a combination of anti-PD-1 and anti-angiogenic agents in patients with advanced mucosal melanoma. The efficacy of this combination could be explained not only by an additive antitumor effect but also by the remodeling of the immuno-suppressive tumor microenvironment to an immuno-stimulatory state by inhibiting VEGF.^20,21^ We could also hypothesize an effect through the KIT pathway, targeted by lenvatinib,^22,23^ but we need further investigations to better understand the mechanisms involved in mucosal melanoma pathogenesis. Our observations, based on a small number of cases (n = 7), need to be supported by Supplementary Data. Currently, combined lenvatinib and pembrolizumab is being evaluated for the treatment of mucosal melanoma in a neoadjuvant setting.^24^

However, this substantial response rate must be weighed against the short-response duration. Indeed, our study showed a lower median DOR (3.1 months) than that observed in the LEAP-004 and phase Ib/II studies (8.3 and 12.5 months, respectively).^14,15^ This difference may be explained by a shorter median follow-up in our study (5.0 months versus 15.3 months in the LEAP-004 study). Nevertheless, we cannot rule out an immune effect of the combination either, as we observed some long responders (DOR ranges from 0.6 to 20.8 months).

The safety profile in the Lenvamel study was consistent with those previously reported for other neoplasms, with the following most common AEs; fatigue, nausea and vomiting hypertension, diarrhea, and hypothyroidism.^14,15,25-28^ However, in our cohort, 81% of patients experienced TRAEs of any grade, and 24% experienced grades 3-4 TRAEs, which is a lower rate than that previously reported.^14,15,25-28^ This lower incidence of TRAEs could be explained by a different regimen dose. Indeed, the starting dose of lenvatinib was 20 mg daily in other studies, even though the mean doses in the LEAP-004 study and our cohort were similar (15 mg vs 15.8 mg daily, respectively). Moreover, we can hypothesize that a substantial proportion of patients already experienced side effects related to prior immunotherapy or other treatments, which may reduce the report of adverse events with this combination. Finally, it is interesting to highlight that no TRAE-related deaths occurred during the follow-up. At data cutoff, 14 (21%) patients had discontinued all treatments because of TRAEs, suggesting that toxicity may be manageable, although requiring a close follow-up. Indeed, previous trials recommended that lenvatinib be commenced at a high dose while managing toxicities by reducing and/or interrupting the dose to maintain lenvatinib efficacy.

This study had some limitations in particular the small number of patients in subgroups and the short follow-up, which may constrain the generalizability of the data. Further research with larger cohorts is needed to provide more robust data. A longer follow-up duration would also offer more comprehensive insights into the long-term efficacy and safety of the treatment. Some other limitations were inherently related to its retrospective design, such as the completeness of safety reporting or the risk of missing data. But, in this multicenter study, all the data were collected using a uniform and standardized CRF across all participating centers. Furthermore, as the proportion of missing data was small, the accuracy of our analyses was high. Thus, these results must be interpreted with caution. Nevertheless, these findings remain interesting in a population where there is a severe lack of therapeutic options.

As anti-PD-1 plus lenvatinib is not approved by the Food and Drug Administration or European Medicines Agency, our data support this treatment option for refractory melanoma, when no study is available after the failure of standard therapies. We also have to keep in mind the negative results of the LEAP-003 study, although not published yet.^29^ Indeed, this study aimed to compare lenvatinib and pembrolizumab versus pembrolizumab as first-line therapy for advanced melanoma. The combination therapy provided no added clinical benefit compared with pembrolizumab alone, justifying that it cannot be a first-line option, and highlighting the need for further investigations to understand resistance mechanisms.

Thus, our study highlights the need for new efficient strategies, particularly in the era of cellular therapies, such as TILs, whose data are promising in melanoma refractory to anti-PD-1.^30^

## Conclusion

The Lenvamel cohort study findings consolidate the encouraging results of the phase II LEAP-004 study by showing an interesting response rate and acceptable safety profile with manageable toxicities in an immuno-refractory and heavily pretreated population with poor prognostic factors. Patients previously treated with the anti-PD-1 and anti-CTLA-4 combination and those with *BRAF* wild-type melanoma seemed to benefit more from this second-line strategy. Our study also provides promising but limited data on patients with advanced mucosal melanoma. All these findings support this strategy for refractory melanoma but also highlight the need for new strategies. However, the resistance mechanisms require further investigation.

## Supplementary material

Supplementary material is available at *The Oncologist* online.

oyae145_suppl_Supplementary_Material

## Data Availability

The data underlying this article will be shared on reasonable request to the corresponding author.
