# Supplementary material for: Real-world outcomes of combined lenvatinib and anti-PD-1 in advanced melanoma: the Lenvamel study, a multicenter retrospective study of the French Group of Skin Cancers (Groupe de Cancérologie Cutanée)
Source: Oncologist. 2024 Jul 2;29(10):e1364–72. doi: 10.1093/oncolo/oyae145 (PMC11449033; doi:10.1093/oncolo/oyae145)
Supplement: oyae145_suppl_Supplementary_Material [file oyae145_suppl_supplementary_material.pdf]

Table S1. Response patterns according to subgroups

Subgroups	All patients, N (%)	ORR, N (%)
**Age, years**		
≥ 65	38 (57)	12 (31.6)
< 65	29 (43)	7 (24.1)
**Primary melanoma,**		
Cutaneous	54 (81)	13 (24.1)
Mucosal	7 (10)	5 (71.4)
Unknown	6 (9)	1 (16.7)
**AJCC 8th edition, N (%)**		
III	2 (3)	0 (0)
IV	65 (97)	19 (29.2)
- M1a	1 (2)	0 (0)
- M1b	7 (10)	5 (71.4)
- M1c	28 (42)	5 (17.9)
- M1D	29 (43)	9 (31.0)
**Active brain metastasis, N (%)**	20 (30)	9 (45.0)
- With concomitant brain stereotactic radiosurgery	14 (70)	5 (25.0)
- Without concomitant brain stereotactic radiosurgery	6 (30)	4 (20.0)
**Number of disease sites N (%)**		
≤3	25 (37)	11 (44.0)
>3	42 (63)	8 (19.0)
**ECOG PS, N (%)**		
0	23 (34)	6 (26.1)
1	35 (52)	11 (31.4)
≥2	9 (14)	2 (22.2)
**Mutations Status, N (%)**		
BRAF V600	13 (19)	2 (15.4)
BRAF wild type	54 (81)	17 (31.5)
NRAS	20 (30)	5 (25)
**LDH, N (%) > ULN ; IC (95%)**		
Normal	33 (49)	9 (27.3)
N > ULN and < 2 x ULN	20 (30)	8 (40)
N ≥ 2 x ULN	6 (9)	0(0)
Unknown	8 (12)	2 (25)
**Number of prior lines of therapy, N (%)**		
1	19 (28)	7 (36.8)
2	18 (27)	6 (33.3)
≥3	30 (45)	6 (20)
**Prior line with Ipilimumab + Nivolumab, N (%)**	58 (87)	19 (32.8)
**BRAF-mutant tumors and prior TT**	12 (18)	1 (8.3%)
**BRAF-mutant tumors without prior TT**	1 (1)	1 (100)
**First line treatment, N (%)**		
Immune checkpoint inhibitors	63 (94)	18 (28.6)
- Anti PD1 in adjuvant setting	13 (19)	6 (46.2)
- Anti PD1 in metastatic setting	24 (36)	2 (8.3)
- Ipilimumab + Nivolumab	26 (39)	10 (38.5)
Targeted therapy	3 (4)	0 (0)
- BRAFi + MEKi in adjuvant setting	1 (1)	0 (0)
- BRAFi + MEKi in metastatic setting	2 (3)	0 (0)
Triplet therapy (Anti PD1 + BRAFi + MEKi)	1 (1)	1 (100)

**Resistance to anti-PD-1, N (%)**		
- Primary resistance in the adjuvant setting	8 (12)	1 (12.5)
- Primary resistance in the metastatic setting	29 (43)	10 (34.5)
- Secondary resistance in the metastatic setting	30 (45)	8 (26.7)
**Anti-PD-1 used in combination with lenvatinib, N (%)**		
Nivolumab	12 (18)	4 (33.3)
- Concomitant intrathecal nivolumab	1 (1.5)	1 (100)
Pembrolizumab	55 (82)	15 (27.3)
**Grade III-IV toxicity, N (%)**		
- Yes	16 (24)	7 (43.8)
- No	51 (76)	12 (23.5)

Table S2. Treatment exposure

**Treatment exposure**	ALL patients N=67
**Lenvatinib**	
Duration, months, median (range)	2.9 (0.1-22.9 months)
Dose intensity, mg/day, mean (SD)	15.8 (3.5)
Initial dose, number of patients, No (%)	
20 mg	44 (66)
14 mg	19 (28)
10 mg	4 (6)
8 mg	0 (0)
Dose reductions, No (%)	
0	49 (73)
1	13 (19)
2	3 (5)
3	2 (3)
Time to first dose reduction, months, median (range)	1.9 (0-8.7)
**Anti-PD-1**	
Duration, months, median (range)	3.3 (0.1-22.9 months)

Table S3. Occurrence of AEs of any grade, grade I-II and grade III-IV, categorized according to the different organs/systems involved

Toxicity Grade / Organs/systems involved	Total Population N=67		
	All grade N (% patients)	Grade I-II N (% patients)	Grade III-IV N (% patients)
**Cutaneous AEs**	14 (21)	14 (21)	0 (0)
**Endocrinologic AEs**	10 (15)	10 (15)	0 (0)
**Gastro-intestinal AEs**	55 (82)	51 (76)	4 (6)
**Hepatic AEs**	6 (9)	5 (7)	1 (2)
**Pulmonary AEs**	1 (2)	1 (2)	0 (0)
**Rheumatologic AEs**	6 (9)	4 (6)	2 (3)
**Cardiovascular AEs**	20 (30)	15 (22)	5 (7)
**Ophtalmological AEs**	1 (2)	0 (0)	1 (2)
**Renal AEs**	9 (13)	8 (12)	1 (2)
**General AEs**	45 (67)	39 (58)	6 (9)
**Haematologic AEs**	5 (7)	4 (6)	1 (2)
**Musculo-skeletal AEs**	11 (16)	11 (16)	0 (0)
